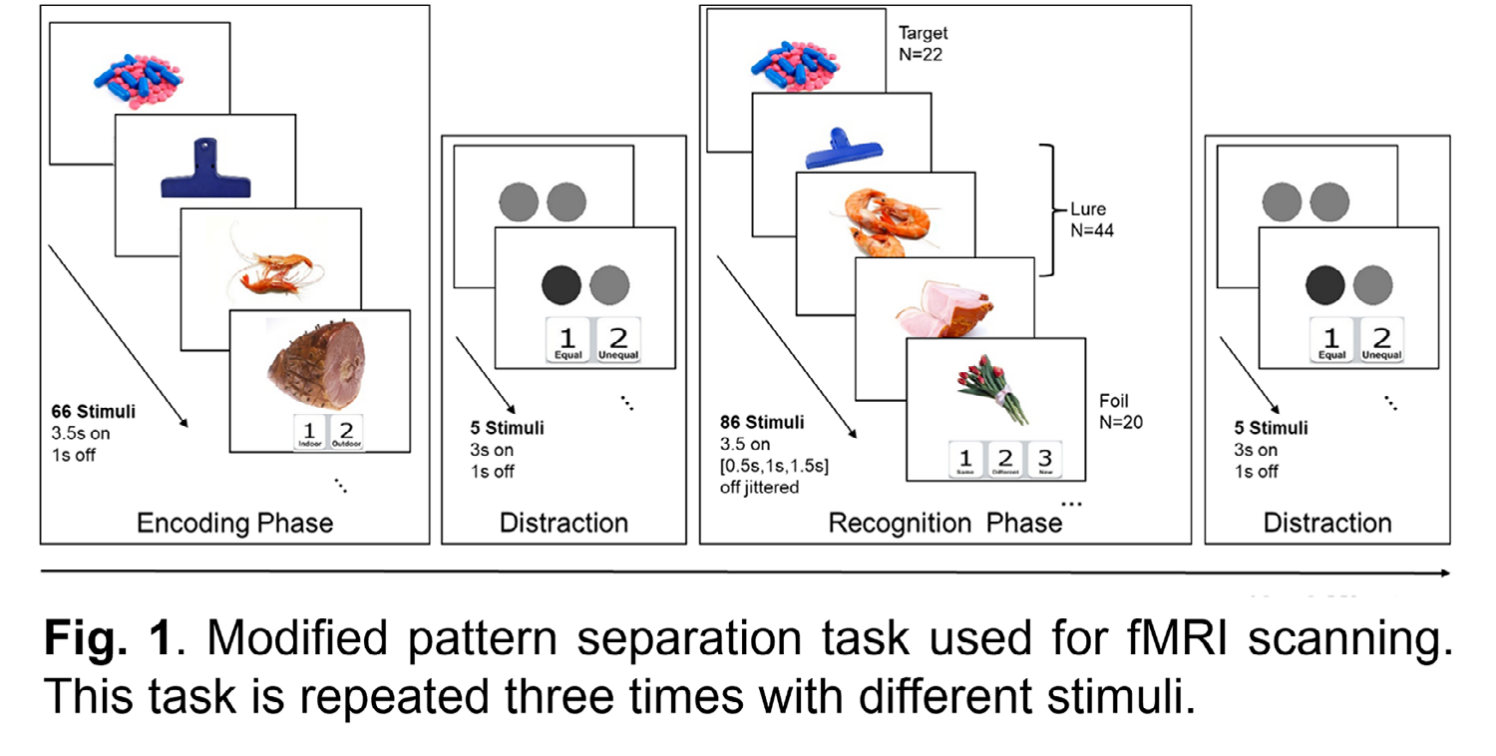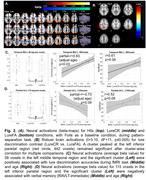# Pattern‐separation induced neural activations in midlife women at risk for AD

**DOI:** 10.1002/alz.086278

**Published:** 2025-01-09

**Authors:** Xiaowei Zhuang, Zhengshi Yang, Dietmar Cordes, Jessica ZK Caldwell

**Affiliations:** ^1^ University of Nevada Las Vegas, Las Vegas, NV USA; ^2^ Cleveland Clinic Lou Ruvo Center for Brain Health, Las Vegas, NV USA

## Abstract

**Background:**

Sex‐specific functional‐brain changes during memory tasks have been reported along the Alzheimer’s disease (AD) continuum. However, mid‐life risk factor effects on memory‐related neural activation remain less clear in women with increased AD risk. Here we examined brain activations during a modified pattern‐separation task and their associations with verbal memory scores in midlife women at risk for AD due to family history.

**Method:**

MRI data were acquired from 12 middle‐aged at‐risk women on a 3‐Tesla Siemens Skyra scanner (34 to 57 years old, mean‐age=50.83). Three task‐functional MRI (fMRI) runs were collected (TR=1.485s, 2mm isotropic, 524 time‐frames) during a modified pattern‐separation task with explicit encoding and recognition phases (detailed in Figure 1).

After preprocessing, a general linear model (GLM) was employed on time‐frames during the recognition phase across three sessions concatenated. Five conditions were modeled, including new‐objects correctly identified as new (Foils), same‐objects correctly identified as same (Hits), similar‐objects correctly identified as similar (LureCR) or falsely identified as same (LureFA), and all other trials. Whole‐brain voxel‐wise analyses were conducted to examine differential activations for lure discrimination (LureCR‐LureFA). The association between significant neural activations and participants' age, task performance, and verbal memory scores (RAVLT) were tested.

**Results:**

Robust neural activations were observed during Hits, LureCR and LureFA conditions (Figure 2(A)).

Significant lure‐discriminative activity (LureCR‐LureFA) was observed in a cluster peaked at left inferior‐parietal, spanning across left‐angular, left‐middle‐temporal, and left‐supramarginal regions (red circle in Figure 2(B)). Neural activations in the left medial temporal region were positively correlated with age (correlation=0.61, p=0.04, Figure 2(C)), and positively correlated with task performance (partial‐correlation=0.83, adjusting for age, p=0.03, Figure 2(C)). A trend‐level negative association was observed between neural activations in the left inferior parietal region and RAVLT‐immediate scores (Figure 2(D)).

**Conclusion:**

In 12 at‐risk women, we observed robust lure‐discriminative activations in the left temporal and inferior parietal regions for during an object pattern‐separation task. Greater neural activation in these regions was evident in participants with higher task accuracies, but older and worse RAVLT performances, potentially indicating a compensatory neural mechanism to achieve high accuracies in a pattern‐separation task in midlife women 10 or 20 years prior to a potential AD onset.